# Perioperative Outcomes of Minimally Invasive Esophagectomy After Neoadjuvant Immunotherapy for Patients With Locally Advanced Esophageal Squamous Cell Carcinoma

**DOI:** 10.3389/fimmu.2022.848881

**Published:** 2022-03-11

**Authors:** Jiahan Cheng, Minzhang Guo, Yushang Yang, Yilin Liu, Weipeng Hu, Qixin Shang, Chuan Li, Liang Xia, Yun Wang, Wenping Wang, Dong Tian, Yong Yuan, Yang Hu, Longqi Chen

**Affiliations:** ^1^ Department of Thoracic Surgery, West China Hospital, Sichuan University, Chengdu, China; ^2^ Institute of Thoracic Oncology, West China Hospital, Sichuan University, Chengdu, China

**Keywords:** esophageal squamous cell carcinoma, neoadjuvant combination of chemotherapy and immunotherapy, neoadjuvant chemotherapy plus radiotherapy, perioperative outcomes, safety and efficiency

## Abstract

**Background:**

Immunotherapy has become a pillar of advanced solid tumors treatment. Patients are more likely to benefit from neoadjuvant immunotherapy compared with traditional neoadjuvant therapy. However, the safety and efficacy of neoadjuvant immunotherapy for the treatment of locally advanced, surgically resectable Esophageal squamous cell carcinoma (ESCC) remain unknown.

**Method:**

ESCC patients who received neoadjuvant treatment following minimally invasive esophagogastrostomy were enrolled from June 2020 to September 2021. The characteristics of neoadjuvant treatment and surgery were investigated to determine the safety and efficacy of the neoadjuvant combination of chemotherapy and immunotherapy (NCI).

**Results:**

A total of 149 patients were included in the study. Patient ratio was 40:109 between NCI and neoadjuvant chemotherapy plus radiotherapy (NCR) groups. No significant difference was found in terms of pathological characteristics, including ypN stage, ypTNM stage, differentiation, lymphovascular invasion, perineural invasion, pathological complete regression and tumor regression score, and these parameters were not correlated with NCI or NCR (all *p*>0.05). Regarding to the operation, the NCI group had less blood loss (49.25 ± 13.47 *vs*. 57.02 ± 47.26, p<0.001), and shorter operation time (247.75 ± 28.28 *vs*. 285.83 ± 52.43, p<0.001) than the NCR group. Additionally, the NCI group demonstrated a lower rate of overall perioperative complications (p=0.003) and grade >2 perioperative complications (*p*=0.042) than the NCR group.

**Conclusion:**

Overall, the findings reported here indicate NCI could result in better outcome and less complications to locally advanced ESCC patients compared with NCR therapy. As a novel therapeutic option, the efficacy and safety of NCI appears to be feasible and safe, while long-term survival data is still needed.

## Introduction

Esophageal cancer, a life-threatening disease, has become the 5th leading cause of death worldwide, of which 5-year survival remains approximately 15–25% due to its high malignant potential and poor prognosis (1–4). Surgery alone is frequently accompanied by high recurrence or metastasis rates leading to poor survival and limited progress among patients with locally advanced esophageal cancer (5, 6). Although substantial improvements in multimodal therapy have been achieved, the all-stage mortality rate of esophageal cancer is still among the highest of all cancer types (7–11). To date, the treatment of esophageal cancer has evolved into a new multidisciplinary process so as to improve long-term survival of patients.

Since the approval of immune checkpoint inhibitors (ICIs), cancer immunotherapy has made an indelible mark in the field of cancer treatment. With widely application of PD-1/PD-L1 and CTLA-4 inhibitors among various indications, these ICI agents has developed a transformative response to advanced solid tumors and unequivocally shown long-term clinical advantages to certain patients over the last decade (12–14). Despite the fact that patients are more likely to benefit from neoadjuvant immunotherapy compared with traditional neoadjuvant therapy (15, 16), the safety and efficacy of immunotherapy for locally advanced, surgically resectable esophageal cancer has not been evaluated comprehensively yet.

Radical resection is typically the mainstay of curative treatment for esophageal cancer; however, it is associated with a high rate of postoperative complications, especially in patients receiving neoadjuvant therapy. Neoadjuvant treatment can cause necrosis, oedema, and adhesion of local tissues, which increases operation difficulty as well as the risk of postoperative complications (17–20). Here, our center conducted a retrospective study to assess the safety and efficacy of neoadjuvant immunotherapy combined with standardized chemoradiotherapy and minimally invasive esophagectomy treatments for patients of locally advanced esophageal squamous cell carcinoma (ESCC).

## Methods

### Patient Samples and Data

Consecutive ESCC patients who received neoadjuvant chemotherapy combined with immunotherapy (NCI) or neoadjuvant chemotherapy combined with radiotherapy (NCR) following minimally invasive esophagogastrostomy (MIE) from January 2020 to May 2021 were retrieved from the esophageal cancer database of West China Hospital. Clinical characteristics, neoadjuvant therapeutic details and operation-related information were exported from our database as well. The study was performed in accordance with the tenets of the Declaration of Helsinki and ethics approval was obtained from the Institutional Review Board and the Ethics Committee of West China Hospital of Sichuan University. Written informed consent were signed from all participants in this study.

### Neoadjuvant Regimens

Patients with following features were recommended to undergo neoadjuvant therapy in terms of the NCCN guidelines of esophageal and esophagogastric junction cancers version 1.2021 (21): (I) ≥18 years old; (II) pathologically confirmed esophageal squamous cell carcinoma; (III) initial diagnosis of clinical TNM stage as T_1_N _+_ M_0_ or T_2-4a_N_0-3_M_0_; and (IV) without severe comorbidities such as active gastrointestinal bleeding, gastrointestinal obstruction, perforation, embolism, shock; (V) noncervical esophagus and (VI) Zubrod-Eastern Cooperative Oncology Group-WHO (ECOG) 0-1 (22). All patients received neoadjuvant chemotherapy, and the recommended regimens included paclitaxel (135–175 mg/m^2^ i.v, d1, q3w) plus cisplatin (75 mg/m^2^ i.v, d1, q3w), fluorouracil (at 750–1000 mg/m^2^ i.v, d1–4, q3w) plus cisplatin (75–100 mg/m^2^ i.v, d1, q3w), etc. Five PD-(L)1 blockades that contained pembrolizumab (200 mg/kg, i.v, q3w), tislelizumab (200 mg, i.v, q3w), camrelizumab (200 mg, i.v, q3w), sintilimab (200 mg, i.v, q3w) and toripalimab (300 mg/kg, i.v, q2w), were applied to neoadjuvant immunotherapy whereas the total radiation dose was 40–50Gy, which was given in 23 fractions of 1.8–2.0Gy each with 5 fractions per week for radiotherapy. Common Terminology Criteria for Adverse Events (CTCAE) version 4.0 was utilized to evaluate the complications caused by neoadjuvant treatments, with grades 2–5 indicating the existence of complications (23).

### Surgical Procedure

ESCC patients were recommended to conduct preoperative assessments to determine the feasibility of the operation after 2-4 cycles of neoadjuvant treatments. McKeown MIE followed by two-field lymph node dissection was used for tumors located in the upper, middle, or lower thoracic segment of the esophagus. Both mechanical and manual sutures were applied with cervical or thoracic anastomosis. Two-field lymph node dissection, including thoracic and abdominal lymph nodes, was conducted for all patients. Based on the N category of the 8^th^ AJCC/UICC TNM stage (24), lymph nodes from resected esophagus were separated and the station of the lymph node was confirmed by surgeons. In addition, the assessment of postoperative complication grades was scored according to the Cavien-Dindo classification (25), with grade ≥3 indicating complications.

### Pathology

Specimens and lymph nodes were examined, after which pathological diagnoses were independently identified by two senior pathologists (Dr. Wang and Dr. Zhou) according to formalin-fixed paraffin-embedded (FFPE) specimens. Pathological information, including tumor invasive depth, lymph node status, metastasis, differentiation, lymphovascular invasion (LVI), perineural invasion and tumor regression score (TRS) was recorded as well. The TNM staging method was based on the ypTNM stage referencing to the 8th edition of the AJCC/UICC staging system (24). TRG was scored by two pathologists on the basis of the presence of residual tumor cells and the fibrosis status (26).

### Statistical Analysis

All statistical analyses were performed with IBM SPSS Statistics version 25.0 (SPSS, Chicago, IL, USA). Methods including Student’s t-test, Chi-square test (*χ* ^2^-test), likehood ratio test Cochran-Armitage trend test and Fisher’s exact test were determined in terms of variable statistics. Statistical significance was determined using the two-tailed test, where *p*-value of <0.05 was considered statistically significant.

## Results

### Patient Characteristics

**Figure 1:**
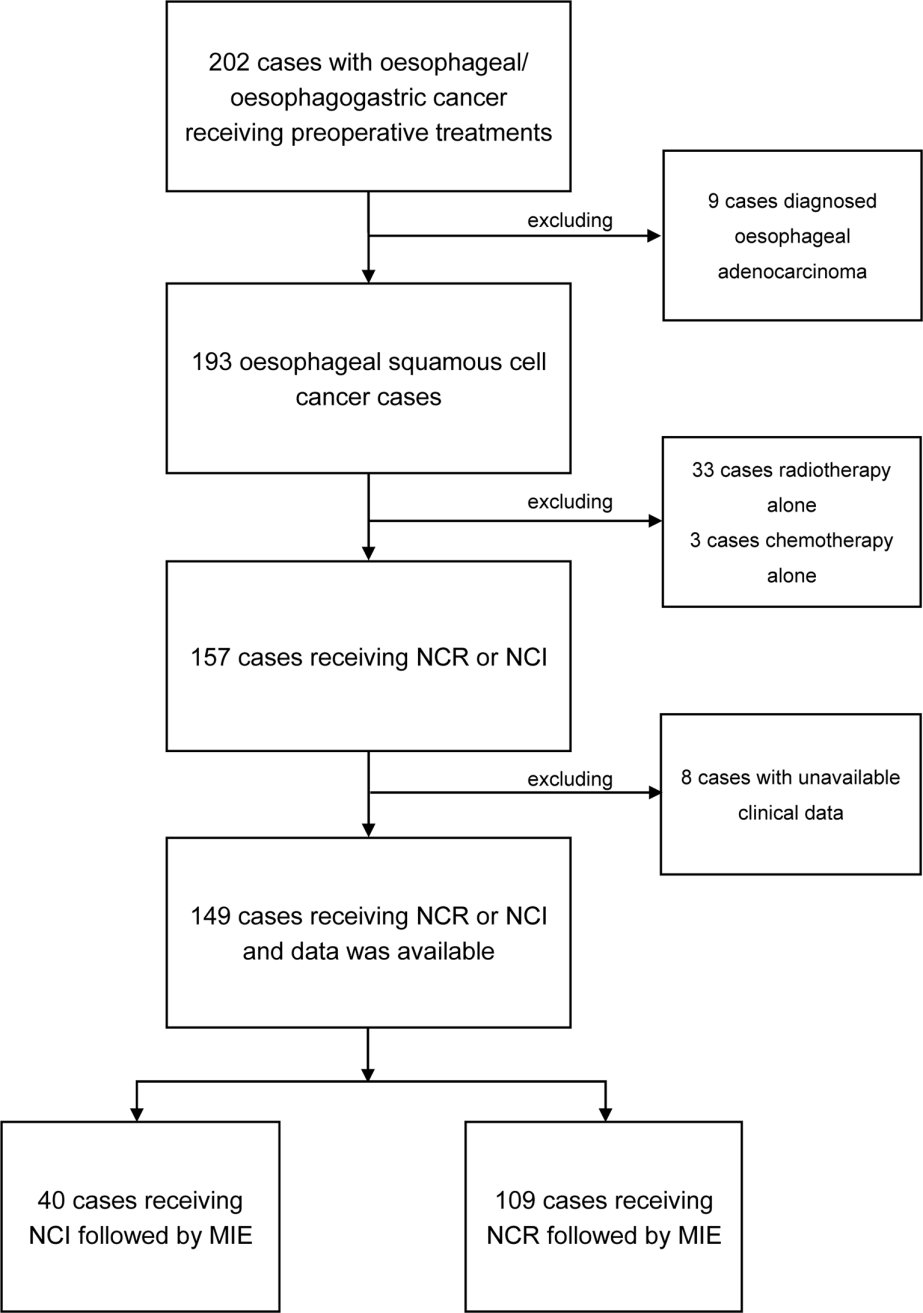
Flow chart for enrolling patients.

From June 2020 to September 2021, a total of 202 patients were pathologically diagnosed with esophageal or esophagogastric cancer, and all received preoperative treatments. Among these patients, 193 were classified as ESCC; 149 received neoadjuvant treatment followed by MIE, 40 received neoadjuvant chemotherapy plus immunotherapy (NCI), and 109 received neoadjuvant chemotherapy plus radiotherapy (NCR). The patient selection process is presented as a flow chart in **Figure 1**. The median ages of the patients classified into the NCI and NCR groups were 64.3 and 62.67, respectively. The NCI group consisted of 30 males (75%) and 10 females (25%), and the NCR group consisted of 93 males (85.3%) and 16 females (14.7%). There were 16 patients with comorbidities in the NCI group and 41 patients with comorbidities in the NCR group. Cardiac diseases accounted for the maximum proportion of comorbidities in the two cohorts. The NCI group and NCR group shared similar percentages of T3 and stage III tumors according to clinical T (cT) stage and clinical TNM (cTNM) stage criteria, and the percentage of tumors located in the middle thoracic segment was >40% in the two groups. The baseline characteristics, which included age, sex, ECOG, cT stage, cN stage, and clinical TNM stage, were not significantly different between the NCI group and NCR group. All the clinical characteristics are shown in **Table 1**.

**Table 1:** The characteristics of two cohorts.

Characteristics	NCI(n=40)	NCR(n=109)	P value
**Age(year)**	64.30 ± 8.88	62.67.7 ± 7.27	0.256
**Sex**			0.141
Male	30(75.0%)	93(85.3%)	
Female	10(25.0%)	16(14.7%)	
**ECOG**			0.950
0	31(77.5%)	85(78.0%)	
1	9(22.5%)	24(22.0%)	
**Comobidity**			0.885
No comobidity	24(60.0%)	68(62.4%)	
Pulmonary Comorbidity	1(2.5%)	7(6,4%)	
Cardiac Comorbidity	9(22.5%)	22(20.2%)	
Renal Insufficiency	2(5.0%)	4(3.7%)	
Diabetes	3(7.5%)	7(6.4%)	
Other comobidity	1(2.5%)	1(0.9%)	
**cT stage**			0.808
T2	2(5.0%)	3(2.8%)	
T3	37(92.5%)	103(94.5%)	
T4	1(2.5%)	3(2.8%)	
**cN stage**			0.075
N0	1(2.5%)	9(8.3%)	
N1	13(32.5%)	54(49.5%)	
N2	25(62.5%)	45(47.0%)	
N3	1(2.5%)	1(0.9%)	
**cTNM stage**			0.778
II	2(5.0%)	9(8.3%)	
III	37(92.5%)	97(89.0%)	
IV	1(2.5%)	3(2.8%)	
**Tumour location**			0.561
Upper-thoracic	12(30.0%)	24(22.0%)	
Middle-thoracic	17(42.5%)	55(50.5%)	
Lower-thoracic	11(27.5%)	30(27.5%)	

### Neoadjuvant Treatments and Adverse Effects

**Figure 2:**
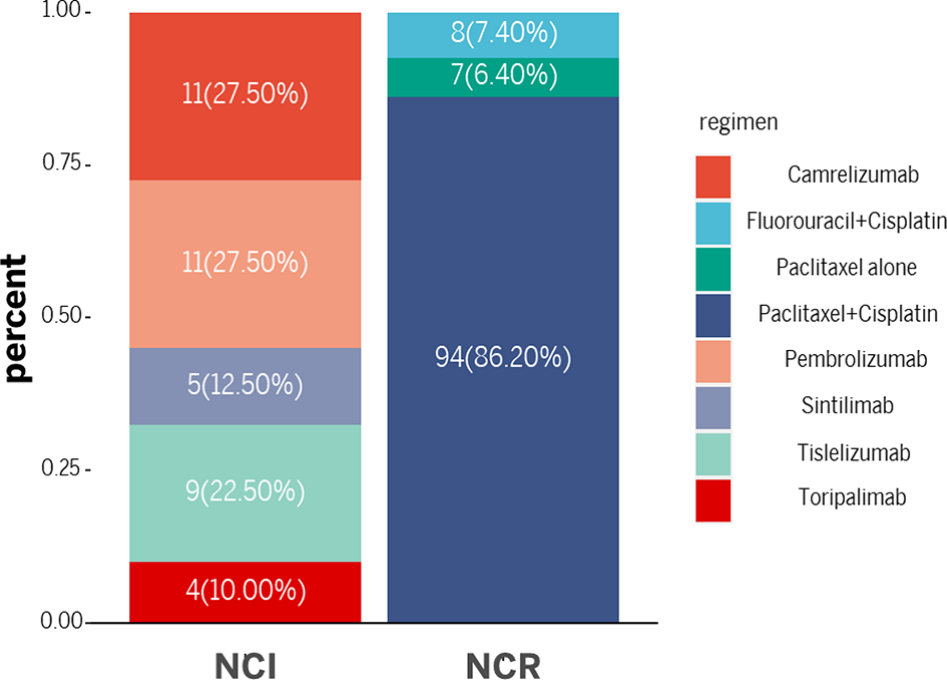
The regimens of neoadjuvant treatments.

Paclitaxel + cisplatin + radiotherapy and fluorouracil + cisplatin + radiotherapy were administered to most patients in the NCR group. Among them, 94 patients received a paclitaxel + cisplatin + radiotherapy regimen, 8 received fluorouracil + cisplatin + radiotherapy and 7 received paclitaxel alone + radiotherapy. In the NCI group, the immunotherapy drugs consisted of PD-1 blockades. Eleven patients received pembrolizumab, 9 received tislelizumab, 11 received camrelizumab, 5 received sintilimab, and 4 received toripalimab. Paclitaxel + cisplatin or fluorouracil + cisplatin was also administered as neoadjuvant chemotherapy in the NCI group. There is no significance on the cycles of neoadjuvant treatment between two groups (2.13 ± 0.76 *vs*. 2.00 ± 0.67, p=0.33). **Figure 2** and **Table 2** demonstrated the regimen details.

**Table 2:** The regimens of neoadjuvant treatments.

Regimen	NCI^#^(n=40)	NCR(n=109)
Pembrolizumab	11(27.5%)	–
Tislelizumab	9(22.5%)	–
Camrelizumab	11(27.5%)	–
Sintilimab	5(12.5%)	–
Toripalimab	4(10.0%)	–
Paclitaxel + cisplatin +radiotherapy	–	94(86.2%)
Fluorouracil + cisplatin +radiotherapy	–	8(7.4%)
Paclitaxel alone +radiotherapy	–	7(6.4%)

^#^Chemotherapy regimens of NCI group also used paclitaxel + cisplatin or fluorouracil + cisplatin regimen.

The incidence of adverse effects in the NCI group and NCR group was 7.5% (3/40) and 5.5% (6/109), respectively, but this difference was not significant (Chi^2 =^ 0.205, *p*=0.657). In addition, two patients experienced grade 2 complications and one experienced grade 3 complications in the NCI group, whereas 4 patients experienced grade 3 adverse effects and 2 experienced grade 2 complications in the NCR group. Additionally, the CTCAE grade distribution was not statistically significant (Chi^2^ = 1.214, *p*=0.576). Other characteristics of adverse effects was presented in **Table 3**.

**Table 3:** The adverse effects of neoadjuvant treatments.

Characteristic	NCI(n=40)	NCR(n=109)	P value
**Adverse effects**			0.702
Yes	3(7.5%)	6(5.5%)	
No	37(92.5%)	103(94.5%)	
**CTCAE grade^#^**			0.576
Grade I	37(92.5%)	103(94.5%)	
Grade II	2(5.0%)	2(1.8%)	
Grade III	1(2.5%)	4(3.7%)	
Grade IV	0	0	
Grade V	0	0	

^#^CTCAE means Common Terminology Criteria for Adverse Events.

### Surgical Outcomes

All patients received McKeown MIE and two-field lymph node dissection by experienced surgeons after finishing neoadjuvant regimens. No significant difference was found in terms of the pathological characteristics, including ypT stage, ypN stage, ypTNM stage, differentiation, LVI, perineural invasion TRS grade and pathological complete regression (pCR), and these parameters were not correlated with NCI or NCR (all *p*>0.05).

Blood loss and operation time presented significant differences between the NCI and NCR groups (all *p*<0.001). However, there was no significant difference in the other operation-related parameters, including hospital stay after operation, lymph node dissection number, LN resection station, number of positive LNs, interval between neoadjuvant therapy and surgery, chest drainage duration and chest drainage volume (all *p*>0.05). The other details of the operative outcomes are summarized in **Table 4**.

**Table 4:** The outcomes of operation.

Characteristic	NCI(n=40)	NCR(n=109)	P value
**ypT stage**			0.528
** T0**	18(45.0%)	53(48.6%)	
** T1**	10(25.0%)	17(15.6%)	
** T2**	9(22.5%)	25(22.9%)	
** T3**	3(7.5%)	14(12.8%)	
**ypN stage**			0.492
** N0**	20(50.0%)	44(40.4%)	
** N1**	15(14.8%)	40(36.7%)	
** N2**	4(6.7%)	21(19.3%)	
** N3**	1(2.5%)	4(3.7%)	
**ypTNM stage**			0.594
** I**	20(50.0%)	43(39.4%)	
** II**	0 (0%)	1(0.9%)	
** III**	19(47.5%)	61(56.0%)	
** IV**	1(2.5%)	4(3.7%)	
**Differentiation**			0.368
Grade1	1(2.5%)	0(0%)	
Grade2	4(10.0%)	16(14.7%)	
Grade3	11(27.5%)	29(26.6%)	
Grade4	24(60.0%)	64(58.7%)	
LVI^*^			1.000
Yes	2(5.0%)	6(5.5%)	
No	38(95.0%)	103(94.5%)	
**Perineural invasion**			0.121
Yes	1(2.5%)	14(12.8%)	
No	39(97.5%)	95(87.2%)	
**TRS^#^**			0.820
TRS0	18(45.0%)	53(48.6%)	
TRS1	6(15.0%)	11(10.1%)	
TRS2	14(35.0%)	32(29.4%)	
TRS3	2(5.0%)	13(11.9%)	
**pCR**	15(37.5%)	40(36.7%)	0.928
**Blood loss**	49.25 ± 13.47	57.02 ± 47.26	**<0.001**
**Operation time**	247.75 ± 28.28	285.83 ± 52.43	**<0.001**
**Stay after operation**	14.58 ± 11.17	12.87 ± 10.16	0.502
**LNs resection number**	23.15 ± 6.55	22.17 ± 6.36	0.957
**LNs resection station**	12.56 ± 3.13	10.18 ± 2.72	0.30
**LNs positive number**	1.23 ± 1.87	1.51 ± 2.03	0.420
**Interval between neoadjuvant therapy and surgery**	67.83 ± 22.57	80.03 ± 31.78	0.205
**Chest drainage duration**	8.680 ± 4.41	9.75 ± 5.79	0.152
**Chest drainage volume**	3315.00 ± 2767.17	3552.29 ± 1318.85	0.158
**30-day mortality**			0.564
Yes	0(0%)	3(2.8%)	
No	40(100%)	106(97.2%)	
**90-day mortality**			0.325
Yes	0(0%)	5(4.6%)	
No	40(100%)	104(95.4%)	

^*^LVI means lymphovascular invasion and ^#^TRG means tumor regression grade. pCR means pathological complete regression. The bold values means the difference has statistical significance.

**Figure 3:**
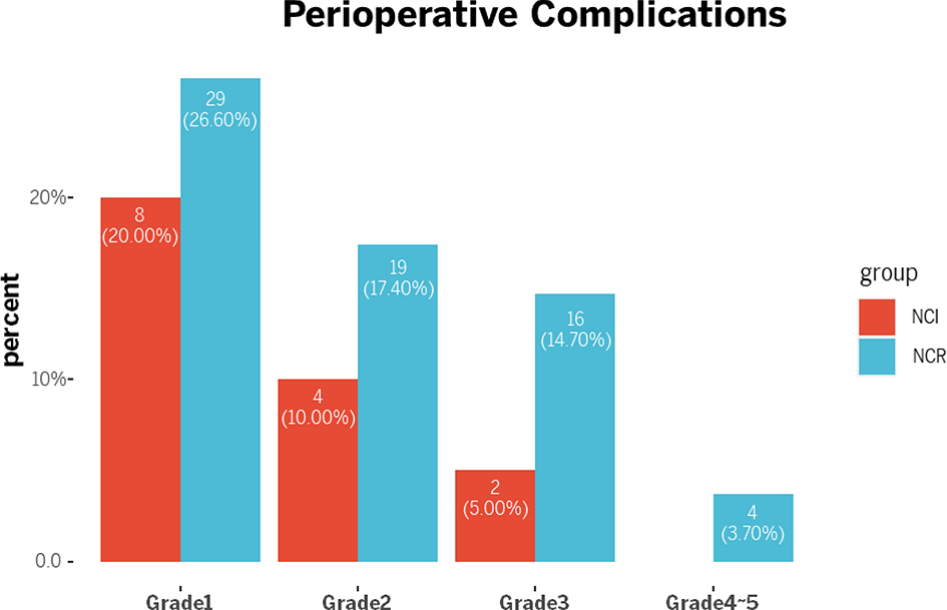
Perioperative complications.

**Figure 4:**
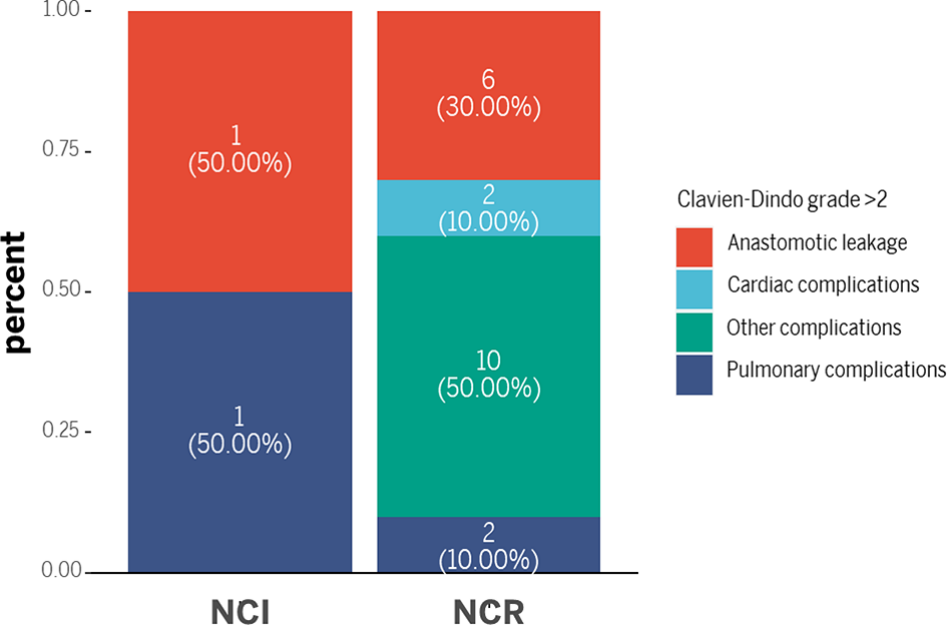
The classification of postoperative complications.

Although the NCI group and NCR group showed no significant differences in 30-day mortality and 90-day mortality (all *p*>0.05), cases of 30-day mortality and 90-day mortality only occurred in the NCR group. According to Clavien-Dindo grade, not only the overall perioperative complications demonstrated a statistical significance (p=0.003), but also a significant difference existed in the term of grade >2 perioperative complications between the two groups, and grade >2 perioperative complications were correlated with different preoperative treatments (*p*=0.042) (**Table 5**). **Figure 3** showed the overall of perioperative complications. In addition, the type of postoperative complications is summarized in **Table 6**. Among them, the NCR group had a higher rate of anastomotic leakage and pleural effusion than the NCI group. **Figure 4** presented the percentage of different CD>2 postoperative complications.

**Table 5:** Perioperative complications.

Characteristic	NCI(n=40)	NCR(n=109)	P value
**Clavien-Dindo grade**			**0.003**
**Without complications**	26(65.0%)	41(37.6%)	
Grade1	8(20.0%)	29(26.6%)	
Grade2	4(10.0%)	19(17.4%)	
Grade3	2(5.0%)	16(14.7%)	
Grade4	0(0%)	1(0.9%)	
Grade5	0(0%)	3(2.8%)	
**Complications**			**0.042**
Yes (CD>2)**^#^**	2(5.0%)	20(18.3%)	
No	38(95%)	89(81.7%)	

^#^CD>2 means Clavien-Dindo grade >2. The bold values means the difference has statistical significance.

**Table 6:** The classification of postoperative complications (CD>2).

Complication type	NCI(n=2)	NCR(n=20)
Anastomotic leakage	1(50.0%)	6(30.0%)
Pulmonary complications	0	2(10.0%)
Cardiac complications	0	2(10.0%)
Wound infection	0	0
Other complications	1(50.0%)	10(50.0%)

## Discussion

Neoadjuvant therapy is considered as a potential approach to prolong postoperative survival of patients with radical resection. Another presumable advantage is to convert inoperable patients with undistinguished tumor boundaries or unresectable lesions to extent of favorable radical resection condition. Preliminary studies suggested immune therapy combined with chemotherapy exhibited the same objective response rate (ORR) as traditional neoadjuvant chemoradiotherapy (27–30). In our study, the NCI group also had the same objective response rate as the NCR group. In addition, the NCI group showed an advantage in perioperative safety such as less blood loss, shorter operation time and a lower rate of grade >2 perioperative complications. NCI is likely to become the balance point between surgery and neoadjuvant treatment, as it can help to increase surgical safety, and at the same time keep the same response rate.

Responses and surgical safety of NCI for patients who diagnosed with gastroesophageal junction adenocarcinoma and underwent the Ivor Lewis operation was reported for the first time by Daniela Molena et al (31, 32). This observation mentioned no significantly difference of perioperative safety between neoadjuvant immunotherapy combined with chemoradiotherapy and traditional neoadjuvant therapy. Likewise, no significant difference was found in 30-day readmission rate and mortality, suggesting neoadjuvant immunotherapy caused perioperative unsafety concerns as well. In our study, most of the tumors location were in the middle thoracic segment of the esophagus and all patients underwent three-incision esophagectomy *via* minimally invasive approach. In our research cohort, NCI and NCR shared the same post pathological characteristics including yp stage, TRS grade and lymphovascular/perineural invasion status. However, NCR’s response to neoadjuvant radiotherapy can contribute to both operation difficulty and intraoperative risk regarding to thoracic tissue injury/edema and pleural adhesions caused by 40-50.4Gy (18). Evidences in this study demonstrated that NCI can result in a safer perioperative period, and also avoid the radiation damage from radiotherapy, thereby reducing the difficulty of the operation.

However, Verdict is still out on long-term prognosis of two neoadjuvant treatment. Previous studies clinically indicated neoadjuvant therapy barely change the recurrence outcome of esophageal cancer patients, mainly occurred with distant metastasis (4–6). It indeed reduced local recurrence rate, but neoadjuvant therapy presented less effective control on distant metastasis. This pattern of recurrence or metastasis may be attributed to minimal residuals disease, which also explained why neoadjuvant therapy was significantly effective, but the long-term survival of patients remained unsatisfactory. Furthermore, local radiotherapy and/or traditional chemotherapy may not show effects on such small residuals or metastases. Although immunotherapy may bring about alterations in treatment guidelines, the pattern of esophageal cancer recurrence after neoadjuvant immunotherapy remains unknown so far. Thus, further investigation will continue to include more patients and track long-term prognosis.

In this study, only patients who had received immunotherapy followed by surgery resection were included for evaluation. Of note, some participants failed a response to neoadjuvant and thus missed the radical surgery opportunity because of tumor progression. To address these patients, novel immunotherapeutic targets and candidate drugs are urgently needed to overcome current treatment bottleneck. Meanwhile, newly tumor microenvironment detection and prediction method of preoperative patients can provide optimal immune adjuvant regimen to achieve promising treatment improvement (33). Previous studies initially have confirmed complementary and synergistic relationship between immunotherapy and other anti-cancer treatments, which presented a therapeutic effect of “1 + 1 > 2” (12–15). Underlying combined treatment models should therefore be explored in esophageal cancer to reach better survival time in patients.

Our study contains some limitations. First of all, this study is a retrospective analysis with relatively small sample size of patients treated with immunotherapy, and thus, the statistical comparisons may be underpowered. Additionally, all patients in this study had received anti-PD-1 treatment and underwent three-incision esophagectomy for middle ESCC, so that our conclusions may not be applicable to other surgical methods or alternative neoadjuvant treatment models.

## Conclusion

Overall, the findings reported here indicate NCI could result in better outcome and less complications to late-stage ESCC patients compared with NCR therapy. As a novel therapeutic option, the efficacy and safety of NCI appears to be safe and well tolerated, while long-term survival data is still needed to provide guidance to clinicians.

## Data Availability Statement

The original contributions presented in the study are included in the article/supplementary material. Further inquiries can be directed to the corresponding authors.

## Ethics Statement

The studies involving human participants were reviewed and approved by the Institutional Review Board and the Ethics Committee of West China Hospital of Sichuan University. The patients/participants provided their written informed consent to participate in this study.

## Author Contributions

Conception and design: LQC, YH, and YY. Administrative support: LQC, YH, and YY. Provision of study materials or patients: WPH, QXS, CL, LX, JHC, YW, and WPW. Collection and assembly of data: JHC, MZG, YSY, and YLL. Data analysis and interpretation: All authors. Manuscript writing: All authors. All authors contributed to the article and approved the submitted version.

## Funding

This study is supported by the National Natural Science Foundation of China (Grant No. 82000514) and key projects of Sichuan Provincial Department of science and technology (Grant No. 2021YFS0233).

## Conflict of Interest

The authors declare that the research was conducted in the absence of any commercial or financial relationships that could be construed as a potential conflict of interest.

## Publisher’s Note

All claims expressed in this article are solely those of the authors and do not necessarily represent those of their affiliated organizations, or those of the publisher, the editors and the reviewers. Any product that may be evaluated in this article, or claim that may be made by its manufacturer, is not guaranteed or endorsed by the publisher.

## Abbreviations

ESCC, Esophageal squamous cell carcinoma; ICIs, Immune checkpoint inhibitors; NCI, Neoadjuvant combination of chemotherapy and immunotherapy; NCR, Neoadjuvant chemotherapy plus radiotherapy; LVI, Lymphovascular invasion; TRS, Tumor regression score; pCR, Pathological complete regression; MIE, Minimally invasive esophagogastrostomy; ECOG, Zubrod-Eastern Cooperative Oncology Group-WHO; CTCAE, Common Terminology Criteria for Adverse Events; cT, Clinical T stage; cTNM, Clinical TNM stage; ORR, Objective response rate.
